# Proceedings from the CIHLMU 2022 Symposium: “Availability of and Access to Quality Data in Health”

**DOI:** 10.1186/s12919-023-00270-1

**Published:** 2023-08-17

**Authors:** Sabita Tuladhar, Kimothy Mwamelo, Christina Manyama, Dorothy Obuobi, Mario Antunes, Mulatu Gashaw, Monica Vogel, Harinee Shrinivasan, Kashung Annie Mugambwa, Isabella Korley, Guenter Froeschl, Lisa Hoffaeller, Sarah Scholze

**Affiliations:** 1 Teaching & Training Unit, Division of Infectious Diseases and Tropical Medicine, University Hospital, LMU, Munich, Germany; 2 Center for International Health, Ludwig-Maximilians-Universität, Munich, Germany

**Keywords:** Data availability, Data ownership, Data quality, Data use, Data security, Low and middle-income countries, Opportunities, Challenges, Privacy, Big Data, Partnerships, Healthcare

## Abstract

Data is an essential tool for valid and reliable healthcare management. Access to high-quality data is critical to ensuring the early identification of problems, the design of appropriate interventions, and the effective implementation and evaluation of health intervention outcomes. During the COVID-19 pandemic, the need for strong information systems and the value of producing high-quality data for timely response and tracking resources and progress have been very evident across countries. The availability of and access to high-quality data at all levels of the health systems of low and middle-income countries is a challenge, which is exacerbated by multiple parallels and poorly integrated data sources, a lack of data-sharing standards and policy frameworks, their weak enforcement, and inadequate skills among those handling data. Completeness, accuracy, integrity, validity, and timeliness are challenges to data availability and use. “Big Data” is a necessity and a challenge in the current complexities of health systems. In transitioning to digital systems with proper data standards and policy frameworks for privacy protection, data literacy, ownership, and data use at all levels of the health system, skill enhancement of the staff is critical. Adequate funding for strengthening routine information systems and periodic surveys and research, and reciprocal partnerships between high-income countries and low- and middle-income countries in data generation and use, should be prioritized by the low- and middle-income countries to foster evidence-based healthcare practices.

## Introduction

William Edward Deming famously said, “In God we trust. All others must bring data”. Access to high-quality data, whether at the individual, sub-national, national, regional, or global level, is essential to ensure the early identification of problems, the design of appropriate interventions, and the effective implementation and evaluation of the health intervention outcomes. However, data availability, accessibility, and quality, particularly in healthcare, remain a major challenge in multiple contexts, with numerous fragmented data sources and data collection methods, even within a country [1]. This challenge is exacerbated by a multitude of parallel and poorly integrated data sources. Even when data is readily available, it may not always be accessible or in a useful format, often with complex and unclear procedures required by relevant authorities in order to gain access. Data security, confidentiality, and the implementation of legal frameworks and policies are other important issues that further restrict access to high-quality data.

Assuming adequate governance, improvements in the quality, quantity, storage, and analysis of health data could represent key pathways to significant improvements in the functioning of the health system and population health. In low- and middle-income settings in particular, which are still experiencing lower population health outcomes today, the effective use of scarce resources needs to be improved. Recent developments in new methods for collecting, analyzing, and applying data on an unprecedented scale—the so-called “Big Data approach”—offer hope and may allow bridging the gap between healthcare delivery and population health, thereby improving many health outcomes. The term “Big Data” is used for data sets of such a grand scale that it makes it difficult for an organization to manage, analyze, and extract value from them using conventional methods and systems [2]. After originally being described with the three dimensions of high volume, velocity, and variety [3], more unique properties have been added over the years to describe and understand the true nature of Big Data and its implications [4].

Despite the progress in recent years, high-quality data are not routinely collected in all settings, important health challenges are not adequately monitored, and effective interventions are not being delivered to people in need. This affects policies and programs and, consequently, the health of entire populations. The CIH^LMU^ Center for International Health 2022 Symposium explored the availability of and access to high-quality data in healthcare from a variety of perspectives. Public health, healthcare, policy, and research professionals from a variety of backgrounds were brought together to discuss key challenges and highlight potential solution strategies.

## Methods

### Institutional framework

Since 2012, the CIH^LMU^ Center for International Health in Munich, Germany, has been organizing an annual symposium series on global health topics. This scientific series, which is also part of the study curriculum for students in the Ph.D. Medical Research-International Health (Ph.D. MR-IH) and M.Sc. International Health (M.Sc. IH) programs, bring together experts and interested audiences in the field of global and international health. The symposium on “Availability of and Access to Quality Data in Health” was the 18th of its kind in this series, organized and conducted entirely by Ph.D. MR-IH and M.Sc. IH students. The conception, organization, and delivery of the symposium not only provide students with the opportunity to gain experience in organizing an international scientific event but also to collaborate with experts in the field. For a successful symposium organization, students must obtain European Credit Transfer System (ECTS) points.

### Organization committee

The organizing committee for the 2022 CIH^LMU^ symposium consisted of six Ph.D. MR-IH candidates and four M.Sc. IH students. The group was facilitated by the Ph.D. MR-IH and M.Sc. IH program coordinators and benefited from a series of symposium preparation and project management workshops organized by CIH^LMU^.

### Topic selection

The COVID-19 pandemic has demonstrated the importance of high-quality data and strong health information systems that provide timely, reliable, and actionable data. During the pandemic, countries’ significant resources were driven towards building resilient health systems, including strengthening information systems to provide high-quality data on the number of individuals tested, positive and recovered cases, deaths, quarantine cases, beds, intensive care units, and ventilators, COVID-19 vaccination rates, research and development of treatments, and secondary effects of COVID-19 on population health. Availability of and access to quality data was therefore chosen as the symposium topic by the Ph.D. MR-IH candidates, who recognize the demand for high-quality data in the health sector and the need to strengthen national data systems in low- and middle-income settings, not only in the face of a pandemic but also to improve health outcomes for populations.

### Symposium delivery

The symposium was held online on March 18, 2022, using the ‘Zoom Meetings’ platform. Registration was free of charge and open from February 8 to March 15, 2022. More than 51 professionals from around the world attended the scientific event, moderated by Ms. Mwamelo and Ms. Mugambwa. The symposium was divided into opening, technical, and closing sessions.

In the opening session, Ms. Manyama, chair of the symposium organizing committee, welcomed all participants, and Dr. med. Froeschl, CIH^LMU^ Board Member and Head of the Teaching and Training Unit at the Department of Infectious Diseases and Tropical Medicine delivered an inaugural speech. In the technical sessions that followed, the presentations by the four speakers each lasted 30 min, followed by a 15-min ‘Question & Answer session, during which participants’ questions, collected either via chat or verbally, were answered. The four speakers’ presentations were followed by a panel discussion with experts in the field of health informatics, epidemiology, and artificial intelligence. Questions from the participants were included in the discussion. Ph.D. MR-IH and M.Sc. IH students of the organizing committee also shared their research projects in five-minute presentations. At the end of the event, Mr. Antunes, co-chair of the symposium organizing committee, gave the closing remarks. Symposium participants holding a medical license were eligible for 7 Continuous Medical Education credit points from the Bavarian Medical Chamber. Certificates of attendance were provided upon request.

## Technical session

### “Data in low- and middle-income countries” Chacha Mangu, MD, Senior Researcher, Epidemiologist, and Head of the Unit of the Epidemiology and Operational Research, National Institute for Medical Research, Mbeya Medical Research Center, Mbeya, Tanzania, and appointed member of several technical committees in Tanzania

#### Overview

Health information systems (HISs) are crucial in addressing health challenges and improving health-service delivery in resource-limited countries. In this regard, the open-source web-based District Health Information Software (DHIS2) provides a solution to one of the biggest challenges of HISs, namely the availability of high-quality and timely data for planning and evaluating the country’s health performance [5].

Data is a representation of facts, concepts, or instructions in a formal manner that is suitable for communication, interpretation, or processing by manual or electronic means [6]. High-quality data serve as a basis for decision-making and program planning across the health system. These decision-making processes include priority setting, annual health planning and budgeting, health resource allocation, and utilization, as well as improvement of service delivery and policies to promote greater utilization of healthcare services and improve health outcomes [7]. Despite improvements, however, data quality remains a concern for healthcare delivery, particularly in low-resource settings. High-quality data are not routinely collected in sufficient detail to allow regular evaluation of levels, trends, and inequities in health outcomes. Key health challenges are not adequately measured and monitored, which also influences the implementation of programs at national and local levels and, consequently, the health status of the population [8].

One of the major issues with data quality in low- and middle-income settings (LMICs) is the insufficient data collection skills of the staff, resulting in, for example, repetition, incompleteness, or invalid data that cannot be used [1]. In most LMICs, data producers, those responsible for data collection and management, come from non-healthcare programs or professions, are generally unfamiliar with health issues, and are often not treated as part of the health workforce. As a result, these personnel often lack core competencies in data collection, management, analysis, interpretation, and presentation, which are essential to improving data demand and use. In addition, front-line health workers are typically overworked, responsible for both data-related tasks and service delivery and therefore give less priority to data collection or outsource tasks to colleagues who are not well trained. Furthermore, there are few capacity-building initiatives, resulting in unqualified staff with a lack of confidence to apply their data collection and use skills in their regular work settings. Low salaries and low motivation further exacerbate the capacity gap in settings without continuous training opportunities.

Although the importance of data is gradually increasing, the current perceived value of data is still considered low in many LMICs [9]. Most often, data are collected only to report to higher authorities, and little or no use is made of the data by those who generate it. Another major data quality issue is the paper-based systems still commonly used in LMICs that do not provide timely, accurate, and reliable information for public health decisions. It is therefore important to transition from paper-based to digital data systems. One of these digital systems is DHIS-2, a free and open-source health management data platform for collecting, validating, analyzing, and presenting data to support decision-making. Many LMICs have made efforts to reform their national health information systems, and countries in Africa and Asia in particular have already implemented DHIS-2 [10, 11]. Digital data are bytes of information that are integrated across different systems and can be processed and interpreted using different technologies for different purposes. Electronic data in LMICs is typically in the form of static documents; the majority, especially those originating from hard paper-based sources, are stored in non-modifiable formats such as PDF files. Other files are electronically tangible; they can be viewed, accessed, and updated. However, especially in low-resource settings, a lack of infrastructure and computers, information, communication, and technology illiteracy, as well as connectivity issues, may result in the necessity to manually collect data on paper forms. Moreover, the use of modern technologies for data management also requires qualified personnel, further underscoring the need for capacity-building initiatives.

#### Discussion and conclusions

High-quality data is essential for good decision-making. Policymakers need fit data to make informed decisions for program planning at the national, subnational, and facility levels and to improve the country’s health outcomes. Despite recent developments in improving data management systems in LMICs, these developments are hampered by structural and political issues. Countries are often good at formulating data policies, but they are rarely implemented. In Tanzania, for example, eleven healthcare data policies and guidelines have been developed to guide health systems, but unfortunately, most of them are not applied. Furthermore, projects are often donor-funded, which inevitably affects data collection, analyses, and outputs. This poses a challenge to country ownership and sustainability, as donor interests may not always align with the priorities of local stakeholders. In addition, data producers and users lack core competencies, posing a major challenge to data quality and management. Using the right technology is also critical to ensuring data quality. Technology transfer is necessary to overcome the overreliance on paper-based data systems, which have several limitations such as lack of storage space, security issues, proneness to damage, document transportation, editing problems, high costs, and environmental damage [12]. Building the capacity of those who generate data and improving data utilization at all levels of health systems are critical to evidence-based healthcare management.

### “Data in high-income countries and international settings” Andrews Akwasi Agbleke, Ph.D., co-founder and president of the Sena Institute of Technology, Ghana

#### Overview

The interest and capacity to build international collaborations to pursue research goals and improve health systems are greater than ever, especially in light of technological advances and increasing population mobility, which is leading to health problems of global concern as the cultural and social determinants of health and disease cross borders [13]. Accordingly, international collaboration on data is also on the rise. Most importantly, demand for Big Data is increasing because the dimensions that define Big Data enable a health systems approach in which health priorities and interventions are guided by the analysis of large data sets. However, to support international collaborations, these data should be made accessible to researchers, academics, program managers, and others for translating knowledge into action. One effective way of sharing data is through publishing articles. When research findings are published, they are accessible to scientists in other parts of the world and allow replication of the research in a new setting. In addition to publishing data in articles, presenting research findings at conferences in the form of an oral or poster presentation provides a platform for direct interaction between researchers, funding and regulatory agencies, journals, and academics by enabling face-to-face discussions. In light of the COVID-19 pandemic, webinars have become an important tool for data sharing over the past two years. They allow an international audience to network with each other without having to travel to a specific location, saving costs and providing a future way for data sharing.

In order to share research findings within the international research community, interoperable data systems that enable data exchange within and between different organizations and research institutions are very important to avoid duplication and improve sharing and accessibility. Web-based platforms that provide a number of analytical tools for data access and analysis are now increasingly made available free of charge. Examples of such platforms include– the University of California Santa Cruz (UCSC) genome browser and galaxy browser, or software such as R and Python, etc. More citations, availability of secondary data for research, the collaboration between countries and regions, and the possibility for low-resource institutions to use existing datasets provide opportunities for data sharing and multiplication of impact. However, consistent global procedures for collecting, processing, and analyzing data to control data quality have yet to be found.

Challenges to data access and use are not only prevalent in LMICs, but there are several concerns about data sharing and ownership in HICs too. First, there is controversy over whether the data are owned by the researcher, the research institution, the publisher, the funding agency, or the patient. In HICs, the patient is currently presumed to own the data, requiring the patient’s consent for any use of clinical data. The researcher or institution collecting the data is merely acting as a data custodian. Once the data has been used for the purpose for which it was collected, it must be destroyed or anonymized so that no one can link it to the patient. This is especially important to maintain patient privacy and confidentiality [14]. Another barrier to data sharing is fear-based resistance from researchers. In an increasingly interconnected and interdependent world, competition is also high. Researchers fear that data sharing will lead to more competition and pressure to publish. They also worry that they will not be the first to publish their own data if it is already shared and that they may lose the opportunity to get multiple publications from a single data set [15]. Other challenges to data sharing in HICs include poor data quality, which exacerbates reluctance to share data; data management and security; and cost issues related to storage, transmission, and cloud computing, journal enforcement, and intellectual property [16, 17].

#### Discussion and conclusions

International collaborations on Big Data are becoming more common. Efforts are being made to foster collaboration, and the international community is reaching a point where collaboration is widespread. Collaboration should be dynamic, meaning that both partners—HICs and LMICs—should have the opportunity to contribute equally in their own way to the research that is of interest to them. By fostering equitable collaboration and continuous outreach, capacity is gradually built and developed in the local environment.

### “Future of Data” Samson Kiware, Ph.D., Principal Research Scientist and Head of Advanced Statistics and Mathematical Modeling Unit at the Ifakara Health Institute, Tanzania; Research and Knowledge Management Program Manager at the Pan-African Mosquito Control Association (PAMCA); courtesy Research Assistant Professor at Marquette University, Wisconsin; and Adjunct Professor at the Nelson Mandela African Institution of Science and Technology, Tanzania

#### Overview

The ability to collect and learn from large amounts of data has been a key driver of innovation in recent years. Everything from healthcare to transportation and entertainment is now driven by data and statistics. However, the ability to collect high-quality data, the capacity to derive insights from it, and the skills to translate those insights into change are still not evenly distributed across the globe. That is why there are still differing views on the future of Big Data in low- and middle-income settings. While some policymakers see the approach as linked to a number of challenges and as a potential distraction, others see Big Data as a critical milestone in improving the country’s health systems [18–20]. However, to avoid becoming a distraction, LMICs have to examine the specific applications for which Big Data would be useful and the local capacity to manage and analyze Big Data.

The sources of Big Data in healthcare are diverse and complex, including government agencies, patient portals, research studies, payer records, smartphones, search engines, public records, etc. LMICs face challenges in harnessing the value of Big Data. There are issues with data ownership, data access, privacy, and security, data infrastructure, a lack of technical capacity, and a high dependency on external experts and funding to design, develop, and maintain Big Data systems. While Big Data appears to be a way forward for LMICs, the countries still need to develop and update data access, data sharing, and data use principles; develop and enforce an appropriate governance framework to protect individuals; comply with data sharing agreements, particularly in the context of regional diseases control and eradication initiatives; establish Big Data initiatives in collaboration with local and international partners; build or strengthen a national-level information technology infrastructure; develop transparent and replicable Big Data prediction models, and use well-understood and continuously analyzed algorithm dynamics to avoid potential systematic measurement error [18, 21].

Moreover, the Big Data approach may exacerbate existing difficulties associated with healthcare delivery in resource-constrained environments. In such settings, it may not be practical for overworked frontline health workers to expand their scope to include the collection of what might be considered non-essential data. Other issues arise from privacy and information service security laws, which are often underdeveloped and rarely enforced in LMICs [20].

Although the use of Big Data in LMICs seems particularly complex, it also offers the greatest potential benefits. With Big Data initiatives come opportunities for LMICs to build local capacity, such as by developing Big Data infrastructure that enables them to predict, decide, and provide policy recommendations; strengthen global disease surveillance; scale health systems; improve healthcare delivery; and develop careers in Big Data initiatives. Adapting open-source applications like DHIS-2 where applicable prevents having to reinvent the wheel and saves resources that can be used for other meaningful activities instead of developing new software. Currently, commonly used Big Data analytics include disease modeling, early disease detection, treatment risk prediction, mass disease prevention, more accurate treatment, personalization of patient care, prediction of treatment costs, the discovery of new therapies and medications, improved workforce management, and better customer service [22–24]. Thus, it is crucial for LMICs to invest in Big Data in health initiatives to take advantage of its opportunities.

#### Discussion and conclusions

The reasons for the failure of Big Data initiatives in low- and middle-income settings include a lack of infrastructure, a qualified workforce for data collection and management, and technical expertise for in-country data analysis [25]. However, international organizations, ministries of health, universities, research institutions, funders, and the private sector need to work together to ensure that everyone can benefit from Big Data. Big Data analytics should complement, not replace, traditional public health surveillance methods, and LMICs should learn from previously failed Big Data initiatives, establish and/or strengthen data science programs at local universities and research institutions, and establish interoperability standards to maximize the benefits of Big Data. To address the data security issues prevalent in LMICs and HICs, governments need to enforce laws and policies that must be updated regularly. Furthermore, the data should be made available to anyone who needs it with confidentiality maintained, and the data system should enable policymakers to make timely decisions to correct ongoing health interventions, design new programs, and formulate strategies and policies.

### “Country case study: Data availability and use in the Nepali health sector” Mr. Shiv Lal Sharma, Senior Statistical Officer in the Integrated Health Management Information System Section of the Department of Health Services at Ministry of Health and Population (MOHP), Nepal

#### Overview

Routine health information systems are a crucial tool for LMICs in decision-making at all levels of the healthcare system. The use of routine health information systems saves costs by preventing the need to carry out resource-intensive surveys frequently and has the potential to provide uninterrupted data for healthcare managers and service providers for routine program management. Although the use of routine health information systems is gradually increasing in LMICs, their use to date has been limited due to concerns about quality, accuracy, timeliness, integrity, and completeness related to technical, behavioral, organizational, environmental, and political factors that impede the progress of evidence-based health programs [26].

Nepal has a population of 30 million and became a federal republic in 2009. The new constitution was promulgated in 2017, and the unitary system was devolved into a federalized system that includes one federal, seven provincial, and 753 municipal governments, increasing the need for more disaggregated data for planning local health programs. Nepal has a well-established network of health systems, with over 52,000 female community health volunteers working at the grassroots level and over 9,000 public and private health facilities across the country [27]. Although there are nearly 15 routine management information systems in Nepal, the HMIS and Logistics Management Information System (LMIS) are the two most frequently used systems in the health sector. With the use of the DHIS-2 platform to manage service statistics since 2016–17, more than 7,000 public health professionals, mainly from the public sector, can access HMIS data by logging into the DHIS-2 system [27]. Data collectors, data entry personnel, and data managers have access to process the data, while other service providers and managers can only view and download the data. In addition, service providers and private facilities are given login access to view and download data upon request. LMIS is a quarterly reporting system, but with the introduction of electronic (e-) LMIS, Nepal is now able to monitor health commodity transactions in real time. By using eLMIS, Nepal is, for example, able to track the influx of COVID-19 commodities procured by the government and donated by donors for accountability, which is key for COVID-19 response efforts. Nepal uses commercial software to manage LMIS, which is now operational at 1,631 service delivery points. The major challenges of eLMIS include high maintenance costs, the constant need for staff training, and interoperability with other information systems [28].

Nepal’s health sector faces challenges such as an insufficient number of approved positions for Information Technology and Data Managers in health facilities, as well as for health officials at the district and provincial levels, and the frequent migration of trained staff to areas outside the healthcare sector. Nepal is a mountainous country, and its difficult geography poses a barrier to smooth internet coverage and online reporting. Data quality is another concern, and the MOHP conducts integrated validation checks in DHIS-2, routine data quality assessments, review meetings, and special assessments to improve HMIS and LMIS data quality. Enabling interoperability between the different information systems is a priority but has yet to be achieved. In the new federal structure, cooperation, collaboration, coexistence, and adequate communication are challenges that impact the functioning of health services and the operationalization of information systems. The quality of HMIS data varies by municipality, health facilities, services, and indicator types [29], and data use at all levels is a challenge. Similarly, private sector regulation and increasing reporting, as well as improving overall reporting in public hospitals, are also challenges for Nepal.

#### Discussion and conclusions

The Government of Nepal pursues an open data policy and HMIS data are made available to the public in the form of annual reports. Aggregated data are also made available to students and researchers upon request in MS Excel format. Individuals who have access to HMIS and LMIS have the facility to download data in Excel format. Log-in access is available to all public staff working in the healthcare system and selected staff from the donor community. Access to data processing is, however, restricted to MOHP statistical officers and data managers. Although the reporting rates have improved following the introduction of the DHIS-2 reporting platform and eLMIS, the timeliness of reporting still needs to be improved.

### Panel discussion

The panel discussion focused on sharing expert perspectives on their current research projects as well as insights into common themes that emerged from the previous presentation: the challenges and opportunities of eHealth, access to data, data sharing and security, and international collaboration on Big Data. Among the panelists was Prof. Sundeep Sahay from India, a professor of digitalization in the Department of Informatics at the University of Oslo and a researcher in the field of health information infrastructure design, development, integration, use, and sustainability in LMICs, with a focus on India’s public healthcare system. In addition, Dr. Daniela Koller from Germany joined the panel discussion. She is a research scientist at the Institute for Medical Data Processing, Biometry, and Epidemiology at the Ludwig-Maximilians-University (LMU) Munich and a coordinator of the Munich Network for Health Care Research. The third panelist was Dr. Deogratias Mzurikwao from Tanzania. Dr. Mzurikwao is leading the artificial intelligence department at Villgro Africa in Kenya. Ms. Mwamelo and Ms. Mugambwa from the symposium organization committee moderated the discussion.

Prof. Sahay’s current work on antimicrobial resistance projects aims to examine health inequities within populations by gender, education, wealth, etc. He emphasized the need for a holistic approach to public health and collaboration between public health professionals, medical doctors, and social and computer scientists to address complex social problems. Prof. Sahay believes that the systems theories of the 1960s are still relevant to addressing complex social issues and that efforts must be made to integrate medicine and social sciences with the help of informatics. E-health is a way forward that needs to be well-equipped on the ground with detailed planning to bridge the gap between policy and implementation. Prof. Sahay emphasized the importance of building systems and highlighted that the design process needs to be more adaptive to address global issues such as climate change, antimicrobial resistance, sustainability, etc. There is a need to move away from the narrow traditional information technology domain and incorporate new thinking, ethics, and partnerships. The new partnerships need to perceive LMICs as a place from which innovation emanates rather than treating them as passive recipients of services and providers of data.

Dr. Koller discussed the challenges for healthcare projects in Germany and Europe, which pursue an integrated research approach with service providers and researchers working together. She highlighted the need for a common data language and quality standards for data aggregation and comparison. Current concerns associated with data include the use of different software requiring different data structures that make it difficult to analyze and use data in a meaningful way, as well as data access and security. Many Big Data datasets are available but not easily accessible. Data protection is important, and there are legal procedures for acquiring data and sharing it with third parties. In Germany and many other parts of Europe, data privacy standards are already very high, and the way forward should be to pursue a more open scientific approach to acquiring and sharing data to improve data quality and use. Dr. Koller also reflected on the massive paper data generated on COVID-19 testing in Germany and the need to digitize it for effective use. Participants expressed concerns about whether the current geopolitical environment in Europe could slow digitalization, and the panelists predicted that additional security considerations may indeed slow the pace of digitalization.

Dr. Mzurikwao pointed out that the number of non-communicable diseases in LMICs appears to be lower than in HICs due to limited diagnostic capacity. For example, in Tanzania, with a population of 60 million, there are only five hospitals that can diagnose cancer and only two mammography units in government hospitals. As a result, he said, at least 50 percent of women diagnosed with breast cancer will die from it [30]. On the other hand, he is optimistic that access to screening and diagnostic facilities in Tanzania will improve due to efforts to develop an algorithm for cancer detection using mammography and ultrasound. He also cited examples of the benefits of machine learning-based technology and surveillance for the diagnosis and treatment of sickle cell anemia and rabies, respectively, which are highly prevalent in Africa and India [31]. Dr. Mzurikwao sees great opportunities for LMICs in the area of artificial intelligence and machine learning but also sees the challenge of overcoming health professionals’ fear that machines may take over their job. However, Dr. Mzurikwao is confident that artificial intelligence will enable clinicians to deliver services more effectively and improve coverage. Another concern is the lack of health regulations and clear privacy policies. He believes that a balance needs to be found between regulating and sharing data and that the system should not overregulate data sharing so that LMICs can reap the benefits of the technology. He further insisted on the need for regulations around artificial intelligence innovation to make sure only responsible artificial intelligence solutions that will protect and save humans are applied. Another challenge is the interoperability of data systems. Often, the new devices and the existing database cannot communicate with each other, but advances are being made continuously, and many of the newer machines already allow interoperability.

The panelists shared their perspectives on strengthening international collaboration on data sharing. With the help of technology, there are opportunities to improve data quality for international comparisons while taking into consideration privacy, legal, and ethical frameworks. When developing data collection tools, care should be taken to ensure that commonly agreed-upon standards are met from the outset. The current nature of collaboration needs to be changed to be more reciprocal. LMICs need to drive collaboration to focus on their country-specific problems and create an environment of mutual respect. The results of the collaborations should then be utilized to strengthen national systems.

## Conclusion

Throughout the symposium, it became evident that access to and availability of quality data are still major challenges in low- and middle-income settings. However, data are an important tool for formulating health policy, designing and managing health programs, and delivering high-quality healthcare. Based on high-quality data, informed decisions can be made at inter- and intra-national levels to ensure that marginalized populations also achieve equitable health outcomes. To close the data gap between LMICs and HICs, more emphasis should be placed on data collection methods, systems that enable open data sharing, and sustainable, well-trained human capital. To improve the quality of the data collected, LMICs need to improve the training of those tasked with data collection. Furthermore, the shift from a paper-based to an electronic data storage system (e.g., DHIS-2) has greatly improved geographic access to data for some. However, for data to be fully available and accessible to those who need it, data-sharing policies and an infrastructure to improve interoperability among ministries, agencies, and organizations are needed. Legal and ethical frameworks and policies for data exchange and its standards, personal data protection, and national security must be developed and implemented. The lack of data quality standards for aggregation and cross-national comparison was evident throughout the symposium and seems to affect LMICs and HICs alike. However, LMICs need resources to develop and strengthen their health systems and to design, manage, and use Big Data. Mutual partnerships between HICs and LMICs can further promote.

## Data Availability

Symposium speakers' presentations are available upon request.
